# A first-in-human phase I study of TAS-117, an allosteric AKT inhibitor, in patients with advanced solid tumors

**DOI:** 10.1007/s00280-023-04631-7

**Published:** 2024-02-27

**Authors:** Toshihiko Doi, Shunji Takahashi, Daisuke Aoki, Kan Yonemori, Hiroki Hara, Kosei Hasegawa, Kazuhiro Takehara, Kenichi Harano, Mayu Yunokawa, Hiroyuki Nomura, Tatsunori Shimoi, Koji Horie, Aiko Ogasawara, Shinichi Okame

**Affiliations:** 1 National Cancer Center Hospital East, Kashiwa, Japan; 2 Cancer Institute Hospital of Japanese Foundation for Cancer Research, Tokyo, Japan; 3 Keio University School of Medicine, Tokyo, Japan; 4 Akasaka Sannou Medical Center, Tokyo, Japan; 5 International University of Health and Welfare Graduate School, Tokyo, Japan; 6 National Cancer Center Hospital, Tokyo, Japan; 7 Saitama Cancer Center, Kita-Adachi, Japan; 8 Saitama Medical University International Medical Center, Hidaka, Japan; 9 NHO Shikoku Cancer Center, Matsuyama, Japan; 10 Fujita Health University, Toyoake, Japan

**Keywords:** Advanced solid tumor, AKT inhibitor, First-in-human, Pharmacodynamics, Pharmacokinetics

## Abstract

**Purpose:**

TAS-117 is a highly potent and selective, oral, allosteric pan-AKT inhibitor under development for advanced/metastatic solid tumors. The safety, clinical pharmacology, pharmacogenomics and efficacy were investigated.

**Methods:**

This phase I, open-label, non-randomized, dose-escalating, first-in-human study enrolled patients with advanced/metastatic solid tumors and comprised three phases (dose escalation phase [DEP], regimen modification phase [RMP], and safety assessment phase [SAP]). The SAP dose and regimen were determined in the DEP and RMP. Once-daily and intermittent dosing (4 days on/3 days off, 21-day cycles) were investigated. The primary endpoints were dose-limiting toxicities (DLTs) in Cycle 1 of the DEP and RMP and incidences of adverse events (AEs) and adverse drug reactions (ADRs) in the SAP. Secondary endpoints included pharmacokinetics, pharmacodynamics, pharmacogenomics, and antitumor activity.

**Results:**

Of 66 enrolled patients, 65 received TAS-117 (DEP, *n* = 12; RMP, *n* = 10; SAP, *n* = 43). No DLTs were reported with 24-mg/day intermittent dosing, which was selected as a recommended dose in SAP. In the SAP, 98.5% of patients experienced both AEs and ADRs (grade ≥ 3, 67.7% and 60.0%, respectively). In the dose range tested (8 to 32 mg/day), TAS-117 pharmacokinetics were dose proportional, and pharmacodynamic analysis showed a reduction of phosphorylated PRAS40, a direct substrate of AKT. Four patients in the SAP had confirmed partial response.

**Conclusion:**

Oral doses of TAS-117 once daily up to 16 mg/day and intermittent dosing of 24 mg/day were well tolerated. TAS-117 pharmacokinetics were dose proportional at the doses evaluated. Antitumor activity may occur through AKT inhibition.

**Trial registration:**

jRCT2080222728 (January 29, 2015).

**Supplementary Information:**

The online version contains supplementary material available at 10.1007/s00280-023-04631-7.

## Introduction

In 2020, new cancer cases worldwide were estimated at 19.3 million, and there were nearly 10.0 million deaths from cancer [1]. The global burden of cancer is expected to rise over time, with a projected increase of 47% to 28.4 million cases by 2040. The phosphatidylinositol-3-kinase (PI3K)-v-akt murine thymoma viral oncogene homolog (AKT)-mammalian target of rapamycin (mTOR) signaling pathway regulates numerous cellular functions, including proliferation, differentiation, metabolism, survival, motility, and autophagy [2]. Many types of tumors have altered PI3K/AKT/mTOR signaling. In the 1980s, researchers established a clear link between this pathway and cancer. More recently, this signal transduction network has emerged as one of the most frequently dysregulated pathways in human tumors [3]. The AKT family has three highly conserved isoforms: AKT1/α and AKT2/β, which are ubiquitously expressed, and AKT3/γ, which has a more restricted tissue distribution and is abundant in nervous tissues [2, 4]. AKT activation occurs in many types of cancers, including acute myeloid leukemia, brain, breast, colon, endometrial cancer, gastric, head and neck, lung, melanoma, multiple myeloma, ovarian, pancreas, prostate, and renal cell carcinoma, and it is a key factor in the oncogenic pathway [2, 5–8]. Reportedly, activation of AKT is associated with a poor prognosis and chemotherapy resistance [9–13]. Thus, AKT is expected to be a promising target in PI3K/AKT/mTOR pathway-activated tumors.

Most AKT inhibitors currently in clinical development are considered pan-AKT inhibitors because they inhibit all three isoforms of AKT [14]. There are two types of pan-AKT inhibitors: adenosine triphosphate (ATP)-competitive inhibitors that bind to the active conformation of AKT, thereby exposing the ATP-binding pocket [15, 16]; and allosteric inhibitors that prevent localization of AKT, thereby blocking AKT phosphorylation and activation [17]. Allosteric AKT inhibitors are more specific than ATP-competitive inhibitors, which have a higher off-target effect [18, 19].

TAS-117 (Taiho Pharmaceutical Co., Ltd., Tokyo, Japan) is an AKT inhibitor being developed for the treatment of advanced or metastatic solid tumors. TAS-117 is a highly potent and selective, oral, allosteric pan-AKT inhibitor [20] with strong anti-proliferative activity against multiple tumor cell lines derived from human cancers, including breast, ovarian, gastric, endometrial, and myeloma (data on file, Taiho Pharmaceutical Co., Ltd.). In preclinical studies, oral dosing of TAS-117 in murine xenograft models of gastric cancer (NCI-N87; *HER2* amplification) and breast cancer (KPL-4; *HER2* amplification, *PIK3CA* mutation) has demonstrated single-agent antitumor activity and was well tolerated [20]. TAS-117 has synergistic or additive effects with chemotherapeutic agents both in vitro and in vivo [21]. The toxicity of repeated TAS-117 dosing has been evaluated in rats and monkeys, including 4-week oral toxicity studies. TAS-117-associated toxicity findings common to both species were dysregulation of carbohydrate metabolism and atrophic changes in the lymphatic/hematopoietic organs and adipocytes (data on file, Taiho Pharmaceutical Co., Ltd.). These toxicological changes tended to return to normal at the end of the recovery period.

This phase I clinical study aimed to investigate the safety, pharmacokinetics, antitumor activity, pharmacodynamics, and pharmacogenomics of TAS-117 and determine the recommended dose (RD) for further development.

## Materials and methods

### Patients

Patients were eligible for inclusion in this study if they were ≥ 20 years of age at enrollment, had histologically or cytologically confirmed advanced or metastatic solid tumors with no standard treatment option, had an Eastern Cooperative Oncology Group performance status (ECOG PS) of 0 or 1, were able to take medications orally, had adequate organ function, and had a life expectancy of at least 60 days. The main exclusion criteria were past or current type 1 or type 2 diabetes requiring treatment, retinopathy requiring ophthalmological therapy, past or present cardiac arrhythmia and/or conduction abnormality, major surgery or extended field radiation therapy within the 4 weeks prior to or limited field radiation therapy within the 2 weeks prior to study drug administration, anticancer treatment within 3 weeks prior to study drug administration, treatment with an investigational agent in the 3 weeks prior to study drug administration, unresolved toxicity of grade > 1 attributed to any prior therapies, current oral steroid treatment, or pregnant or lactating. A complete list of exclusion criteria is provided in Supplemental Text 1. All patients provided written informed consent for study participation.

### Study design and treatments

This was a phase I, open-label, non-randomized, dose-escalating first-in-human study to evaluate the safety, pharmacokinetics, antitumor activity, pharmacodynamics, and pharmacogenomics of TAS-117 in patients with advanced solid tumors. The study had three phases: a dose escalation phase (DEP), a regimen modification phase (RMP), and a safety assessment phase (SAP). The treatment cycle on each regimen lasted 21 days. The DEP used an accelerated titration and 3 + 3 design. TAS-117 was administered orally once daily (QD) on an empty stomach (1 h before or 2 h after a meal). Dosing was determined based on a 4-week repeated oral-dose toxicology study in monkeys (data on file, Taiho Pharmaceutical Co., Ltd.); the highest non-severely toxic dose was 1.2 mg/kg/day, which was converted to 3.84 mg/body/day, or 2.4 mg/m^2^. Therefore, the starting dose was determined to be ≤ 2.4 mg/m^2^. At dose level 1, TAS-117 was administered at 2 mg QD (Day − 2), and the dose was increased to 4 mg QD after 2 days (Day 1). The RMP was conducted according to a 3 + 3 design. During this phase, the dose regimen was changed from QD administration to intermittent dosing of daily TAS-117 with 4 days on/3 days off. TAS-117 was administered on an empty stomach. Dose-limiting toxicities (DLTs) were evaluated in Cycle 1 of the DEP and RMP. The maximum tolerated dose (MTD) was defined as the highest dose level at which DLTs were observed in < 33% of patients in Cycle 1. The RD and regimen for the SAP were determined based on the safety, pharmacokinetics, pharmacodynamics, and antitumor activity profile determined in the DEP and RMP. During the SAP, the safety and preliminary efficacy profiles of TAS-117 were assessed at the RD and recommended regimen. Three cohorts were included in this assessment. Cohort 1 included patients with endometrial cancer with a *PIK3CA* mutation; cohort 2 included patients with endometrial cancer with an *AKT1* mutation, *AKT1* amplification, or *AKT2* amplification; and cohort 3 included patients with ovarian clear cell carcinoma.

Blood samples for pharmacokinetic analysis were collected immediately prior to dosing (0 h) and at 0.5, 1, 2, 3, 4, 6, 8, 12, and 24 h post-dose on Day 1 and Day 21 of Cycle 1 for patients in the DEP (QD dosing). For patients who received intermittent dosing regimens, blood was collected immediately prior to dosing (0 h) and at 1, 2, 3, 4, 6, 8, 10, and 24 h post-dose on Cycle 1 Day 1; immediately prior to dosing (0 h) on Cycle 1 Days 3, 4, and 8; and immediately prior to dosing (0 h) and at 1, 2, 3, 4, 6, 8, 10, 24, 48, and 96 h post-dose on Cycle 1 Day 18. Urine samples for pharmacokinetic analysis were collected immediately prior to dosing (single sample collection) and from 0 to 6 h and from 6 to 12 h and 12 to 24 h post-dose on Day 1 of Cycle 1 for both QD and intermittent dosing. Blood samples for pharmacodynamic analysis were collected at pre-dose and 4, 8, and 24 h post-dose on Cycle 1 Day 1, and at pre-dose and 4 h post-dose on Cycle 1 Day 21 (QD dosing) or Cycle 1 Day 18 (intermittent dosing).

The phosphorylated proline-rich AKT substrate of 40 kDa (pPRAS40) level was found to be an indicator of AKT activation, given that PRAS40 is a substrate of AKT [22]. The pharmacodynamic activity of TAS-117 for AKT inhibition was evaluated by the change in the ratio of pPRAS40 (threonine 246) to total PRAS40 in platelet-rich plasma samples before and after TAS-117 administration. The phosphorylation status of PRAS40 in the blood was measured with an electrochemiluminescence detection assay using a Meso Scale Discovery instrument (Meso Scale Diagnostics, Rockville, MD, US). For efficacy, solid tumors were assessed using Response Evaluation Criteria in Solid Tumors (RECIST; version 1.1). Computed tomography scans were evaluated at baseline, every 6 weeks after initiating TAS-117 administration, and at the time of study discontinuation.

Blood samples for pharmacogenomics analyses were collected at baseline (within 7 days of the first study drug administration) for the DEP and RMP and cohort 3 of the SAP. Because SAP cohorts 1 and 2 included patients positive for *PIK3CA,* or *AKT* gene abnormalities, blood samples for patients in these cohorts were collected during the biomarker screening period (i.e., after obtaining consent for pharmacogenomics pre-testing and before obtaining consent for study participation) to confirm eligibility. Thus, additional blood samples were optional for patients who had previously been reliably confirmed to be positive for *PIK3CA* or *AKT* gene abnormalities. Cell-free DNA in blood was analyzed for *PIK3CA* and *AKT1* mutations and *AKT1* and *AKT2* amplifications using droplet digital polymerase chain reaction (PCR). Formalin-fixed paraffin-embedded (FFPE) tumor tissues obtained during surgery or used for definitive diagnoses were collected for pharmacogenomics analyses (required for participants in the SAP, optional for participants in the DEP and RMP). FFPE tumor tissue was evaluated for the presence of *PIK3CA* using PCR and for *AKT1* mutation and *AKT1* and *AKT2* amplifications using droplet digital PCR; messenger RNA levels of *AKT1*, *AKT2*, *AKT3*, *PIK3C2B*, *PHLDA1*, and *ACTB* using real-time reverse transcriptase PCR; levels of AKT2 and pan-AKT protein using immunohistochemistry; and analysis of genomic abnormalities using next-generation sequencing.

This study was initially registered at the Japic Clinical Trials Information (JapicCTI, registered as JapicCTI-152780 on January 29, 2015). Because of the integration of JapicCTI into the Japan Registry of Clinical Trials (jRCT), the study was registered to the jRCT (jRCT2080222728) on January 29, 2015. The registration date remains the same as in the JapicCTI.

### Outcomes

The primary endpoints were the proportion of patients who experienced DLTs in Cycle 1 of the DEP and RMP and the incidence of adverse events (AEs) and adverse drug reactions (ADRs) in the SAP. AEs and ADRs were coded using the Medical Dictionary for Regulatory Activities (MedDRA; version 24.1) and graded according to the National Cancer Institute Common Terminology Criteria for Adverse Events (CTCAE; version 4.03). Secondary endpoints were pharmacokinetic findings, changes in the ratio of phosphorylated PRAS40 (pPRAS40)/total PRAS40 from baseline, efficacy, and pharmacogenomics findings.

### Statistical methods

The sample size for the DEP and RMP was not determined based on statistical considerations. Given that both phases aimed to explore different dose levels based on the occurrence of DLTs, a sample size could not be predetermined. It was assumed that the MTD and pharmacokinetics of TAS-117 could be determined and safety evaluated with a maximum of 54 patients for the DEP and a maximum of 54 patients for each regimen in the RMP. For the SAP, the sample size was determined as 20 patients for each cohort. With a sample size of 20, a null proportion of 5%, and a confidence level of 5%, the power was calculated to be > 80% when an alternative proportion was ≥ 30%.

Regarding the analysis populations, all treated patients included all patients who were enrolled and received at least one dose of TAS-117, and the full analysis set included all patients who were administered the study drug and had data for at least one efficacy endpoint.

Descriptive data were used to summarize safety data and antitumor activity. Pharmacokinetic parameters were derived using non-compartmental analysis and summarized descriptively by dose level, study day, and study phase. The dose proportionality of the maximum plasma concentration (C_max_) and area under the plasma concentration–time curve (AUC) of TAS-117 on Day 1 were assessed using linear regression and power model analyses. The accumulation of TAS-117 was assessed by comparing pharmacokinetic parameters on Day 1 and Day 21 (QD) or Day 18 (intermittent dosing). Pharmacodynamic data were presented using summary statistics of the ratio of pPRAS40/total PRAS40 to baseline values (pre-dose) on Day 1 and Day 18 or 21, by dose level and treatment regimen. Pharmacogenomics data were summarized, and the relevance between biomarkers and efficacy was analyzed using Fisher’s Exact test.

*P*-values < 0.05 were considered significant. Missing data were not imputed, and analyses were performed using all available data. Plasma pharmacokinetic parameters were evaluated using Phoenix^®^ WinNonlin^®^ software (Pharsight Corporation as part of Certara, Princeton, NJ, US, Version 8.1 or 8.3). Statistical analyses were performed using SAS version 9.4 (SAS Institute; Cary, NC, US).

## Results

### Patients

Patients were enrolled between 23 February 2015 and 10 June 2019; the data cut-off date was 31 December 2021. Patient enrollment for each study phase is shown in Supplemental Fig. 1. Twelve patients were enrolled and treated in the DEP and received TAS-117 at dose levels 1 to 4 (4, 8, 16, and 24 mg QD). Ten patients were enrolled and treated in the RMP and received TAS-117 at a dose of either 24 or 32 mg daily with 4 days on/3 days off. The SAP enrolled 16 patients in cohort 1, 7 in cohort 2, and 21 in cohort 3. Patients in the SAP were treated with TAS-117 24 mg daily for 4 days on/3 days off. One patient enrolled in cohort 3 did not receive treatment.

**Fig. 1:**
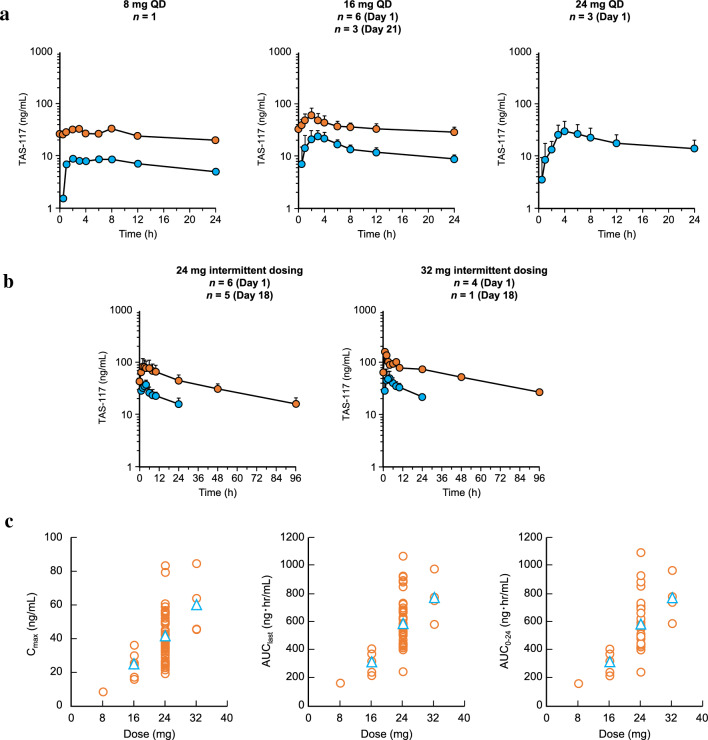
Pharmacokinetics of TAS-117. **a** Mean plasma concentration–time profiles of TAS-117 in the DEP (QD dosing). Data are shown as mean (SD), blue circles represent TAS-117 concentration following Day 1 dosing, and orange circles represent TAS-117 dosing following Day 21. **b** Mean plasma concentration–time profiles of TAS-117 in the RMP (intermittent dosing; 4 days on/3 days off). Data are shown as mean (SD), blue circles represent TAS-117 concentration following Day 1 dosing and orange circles represent TAS-117 dosing following Day 18. **c** Dose proportionality of C_max_, AUC_last_, and AUC_0–24_ across TAS-117 dosing. Open orange circles represent individual values, and open blue triangles represent mean values. *AUC* area under the plasma concentration–time curve, *AUC*_*0–24*_ AUC from time 0 to 24 h, *AUC*_*last*_ AUC up to the last observable concentration, *C*_*max*_ maximum plasma concentration, *DEP* dose escalation phase, *QD* once daily, *RMP* regimen modification phase, *SD* standard deviation

Demographic and background characteristics for all treated patients are shown in Table 1. The median (min, max) age of patients was 59 years (24, 78), and 78.5% were female (100% of patients in the SAP were female). Most patients had ECOG PS of 0 (83.1%), and all were Asian. Nearly all patients had metastatic tumors (98.5%) and histories of surgery (89.2%) and chemotherapy (98.5%). The most common type of cancer among patients was endometrial (35.4%), followed by ovarian (30.8%), other (16.9%), colorectal (9.2%), and finally bladder, breast, gastrointestinal stromal tumor, pancreatic, and uterus (all, 1.5%).

**Table 1:** Patient demographic and background characteristics (all treated patients)

	DEP	RMP	SAP			Total
	*n* = 12	*n* = 10	Cohort 1 *n* = 16	Cohort 2 *n* = 7	Cohort 3 *n* = 20	*N* = 65
Sex						
Male	8 (66.7)	6 (60.0)	0 (0.0)	0 (0.0)	0 (0.0)	14 (21.5)
Female	4 (33.3)	4 (40.0)	16 (100.0)	7 (100.0)	20 (100.0)	51 (78.5)
Age, years						
Mean (SD)	58.7 (14.3)	55.4 (10.6)	59.0 (11.0)	58.7 (14.0)	52.9 (8.3)	56.5 (11.2)
Median (min, max)	62.5 (24, 78)	52.5 (44, 71)	61.0 (28, 75)	67.0 (39, 75)	53.0 (41, 69)	59.0 (24, 78)
ECOG PS						
0	11 (91.7)	6 (60.0)	12 (75.0)	7 (100.0)	18 (90.0)	54 (83.1)
1	1 (8.3)	4 (40.0)	4 (25.0)	0 (0.0)	2 (10.0)	11 (16.9)
Race						
Asian	12 (100.0)	10 (100.0)	16 (100.0)	7 (100.0)	20 (100.0)	65 (100.0)
Type of cancer						
Bladder	0 (0.0)	1 (10.0)	0 (0.0)	0 (0.0)	0 (0.0)	1 (1.5)
Breast	0 (0.0)	1 (10.0)	0 (0.0)	0 (0.0)	0 (0.0)	1 (1.5)
Colorectal	4 (33.3)	2 (20.0)	0 (0.0)	0 (0.0)	0 (0.0)	6 (9.2)
GIST	1 (8.3)	0 (0.0)	0 (0.0)	0 (0.0)	0 (0.0)	1 (1.5)
Pancreas	1 (8.3)	0 (0.0)	0 (0.0)	0 (0.0)	0 (0.0)	1 (1.5)
Ovary	0 (0.0)	0 (0.0)	0 (0.0)	0 (0.0)	20 (100.0)	20 (30.8)
Uterus	0 (0.0)	1 (10.0)	0 (0.0)	0 (0.0)	0 (0.0)	1 (1.5)
Other	6 (50.0)	5 (50.0)	0 (0.0)	0 (0.0)	0 (0.0)	11 (16.9)
Endometrial	0 (0.0)	0 (0.0)	16 (100.0)	7 (100.0)	0 (0.0)	23 (35.4)
Primary tumor						
No	9 (75.0)	6 (60.0)	15 (93.8)	5 (71.4)	19 (95.0)	54 (83.1)
Yes	3 (25.0)	4 (40.0)	1 (6.3)	2 (28.6)	1 (5.0)	11 (16.9)
Metastatic tumor						
No	0 (0.0)	0 (0.0)	0 (0.0)	0 (0.0)	1 (5.0)	1 (1.5)
Yes	12 (100.0)	10 (100.0)	16 (100.0)	7 (100.0)	19 (95.0)	64 (98.5)
Surgical history						
No	3 (25.0)	2 (20.0)	0 (0.0)	2 (28.6)	0 (0.0)	7 (10.8)
Yes	9 (75.0)	8 (80.0)	16 (100.0)	5 (71.4)	20 (100.0)	58 (89.2)
Adjuvant chemotherapy						
No	6 (50.0)	8 (80.0)	6 (37.5)	4 (57.1)	7 (35.0)	31 (47.7)
Yes	6 (50.0)	2 (20.0)	10 (62.5)	3 (42.9)	13 (65.0)	34 (52.3)
Neoadjuvant chemotherapy						
No	12 (100.0)	8 (80.0)	14 (87.5)	7 (100.0)	19 (95.0)	60 (92.3)
Yes	0 (0.0)	2 (20.0)	2 (12.5)	0 (0.0)	1 (5.0)	5 (7.7)
Prior radiation therapy						
No	8 (66.7)	3 (30.0)	12 (75.0)	6 (85.7)	17 (85.0)	46 (70.8)
Yes	4 (33.3)	7 (70.0)	4 (25.0)	1 (14.3)	3 (15.0)	19 (29.2)
Chemotherapy						
No	0 (0.0)	0 (0.0)	0 (0.0)	0 (0.0)	1 (5.0)	1 (1.5)
Yes	12 (100.0)	10 (100.0)	16 (100.0)	7 (100.0)	19 (95.0)	64 (98.5)
Hormonal therapy						
No	12 (100.0)	9 (90.0)	12 (75.0)	7 (100.0)	20 (100.0)	60 (92.3)
Yes	0 (0.0)	1 (10.0)	4 (25.0)	0 (0.0)	0 (0.0)	5 (7.7)
Other prior therapy						
No	10 (83.3)	9 (90.0)	14 (87.5)	7 (100.0)	20 (100.0)	60 (92.3)
Yes	2 (16.7)	1 (10.0)	2 (12.5)	0 (0.0)	0 (0.0)	5 (7.7)

Data are presented as *n* (%) unless otherwise indicated

*DEP* dose escalation phase, *ECOG PS* Eastern Cooperative Oncology Group performance status, *GIST* gastrointestinal stromal tumor, *RMP* regimen modification phase, *SAP* safety assessment phase, *SD* standard deviation

### Safety

In the DEP, TAS-117 was administered QD from dose levels 1 (4 mg) to 4 (24 mg). All patients at dose level 4 experienced grade 2 maculopapular rash requiring study interruption. Thus, the RD was determined to be TAS-117 16 mg QD. In the RMP, intermittent administration (4 days on/3 days off) at dose level 4 (24 mg) and dose level 5 (32 mg) were evaluated. At dose level 5, one of the three patients in whom DLT was evaluated experienced a DLT of grade 3 maculopapular rash during Cycle 1, resulting in treatment interruption. In Cycle 1 and Cycle 2 of dose level 5, two of three patients had grade 3 maculopapular rash, and treatment with TAS-117 was interrupted, and/or a dose reduction was necessary. Given that no DLTs were reported at dose level 4, 24 mg was determined to be the RD for intermittent dosing. A dose of TAS-117 24 mg intermittent administration was evaluated in the SAP.

AEs and ADRs (ADRs with a frequency of ≥ 10%) are shown in Table 2. Nearly all patients experienced any AE (98.5%) and any ADR (98.5%); 67.7% and 60.0% experienced grade ≥ 3 AEs and ADRs, respectively. ADRs with a frequency of ≥ 20% in all treated patients included all-grade nausea, stomatitis, pyrexia, neutrophil count decreased, white blood count decreased, hyperglycemia, pruritus, and maculopapular rash (both all-grade and grade ≥ 3). In total, four patients at dose level 4 (24 mg QD intermittent administration) discontinued because of AEs, which included cellulitis (*n* = 1; serious, not related to treatment), pneumonitis (*n* = 1; serious, related to treatment), rash maculopapular (*n* = 1; not serious, related to treatment), and γ-glutamyltransferase increased (*n* = 1; not serious, related to treatment). One patient (SAP, cohort 1) died because of grade 5 respiratory failure, which was related to the patient’s underlying disease and judged by the study investigator as unrelated to TAS-117 treatment.

**Table 2:** Adverse events and adverse drug reactions (adverse drug reactions with a frequency of ≥ 10%, all treated patients)

	DEP *n* = 12		RMP *n* = 10		SAP *n* = 43		Total *N* = 65	
	All grade	≥ Grade 3	All grade	≥ Grade 3	All grade	≥ Grade 3	All grade	≥ Grade 3
Adverse events								
Any events	11 (91.7)	3 (25.0)	10 (100.0)	7 (70.0)	43 (100.0)	34 (79.1)	64 (98.5)	44 (67.7)
GI disorders	7 (58.3)	0 (0.0)	9 (90.0)	2 (20.0)	35 (81.4)	2 (4.7)	51 (78.5)	4 (6.2)
Diarrhea	3 (25.0)	0 (0.0)	3 (30.0)	1 (10.0)	9 (20.9)	0 (0.0)	15 (23.1)	1 (1.5)
Nausea	2 (16.7)	0 (0.0)	4 (40.0)	0 (0.0)	15 (34.9)	0 (0.0)	21 (32.3)	0 (0.0)
Stomatitis	3 (25.0)	0 (0.0)	8 (80.0)	0 (0.0)	27 (62.8)	1 (2.3)	38 (58.5)	1 (1.5)
Vomiting	3 (25.0)	0 (0.0)	2 (20.0)	0 (0.0)	8 (18.6)	0 (0.0)	13 (20.0)	0 (0.0)
General disorders and administration site conditions	5 (41.7)	0 (0.0)	9 (90.0)	0 (0.0)	25 (58.1)	0 (0.0)	39 (60.0)	0 (0.0)
Pyrexia	1 (8.3)	0 (0.0)	6 (60.0)	0 (0.0)	17 (39.5)	0 (0.0)	24 (36.9)	0 (0.0)
Investigations	6 (50.0)	3 (25.0)	7 (70.0)	3 (30.0)	29 (67.4)	18 (41.9)	42 (64.6)	24 (36.9)
ALT increased	3 (25.0)	0 (0.0)	1 (10.0)	0 (0.0)	8 (18.6)	2 (4.7)	12 (18.5)	2 (3.1)
AST increased	3 (25.0)	1 (8.3)	2 (20.0)	0 (0.0)	8 (18.6)	0 (0.0)	13 (20.0)	1 (1.5)
GGT increased	2 (16.7)	1 (8.3)	2 (20.0)	1 (10.0)	7 (16.3)	6 (14.0)	11 (16.9)	8 (12.3)
Neutrophil count decreased	3 (25.0)	0 (0.0)	3 (30.0)	0 (0.0)	15 (34.9)	9 (20.9)	21 (32.3)	9 (13.8)
WBC count decreased	3 (25.0)	1 (8.3)	3 (30.0)	2 (20.0)	19 (44.2)	5 (11.6)	25 (38.5)	8 (12.3)
Blood AP increased	2 (16.7)	1 (8.3)	1 (10.0)	0 (0.0)	5 (11.6)	0 (0.0)	8 (12.3)	1 (1.5)
Metabolism and nutrition disorders	9 (75.0)	2 (16.7)	7 (70.0)	2 (20.0)	28 (65.1)	9 (20.9)	44 (67.7)	13 (20.0)
Hyperglycemia	4 (33.3)	2 (16.7)	3 (30.0)	2 (20.0)	23 (53.5)	7 (16.3)	30 (46.2)	11 (16.9)
Decreased appetite	5 (41.7)	0 (0.0)	7 (70.0)	0 (0.0)	7 (16.3)	1 (2.3)	19 (29.2)	1 (1.5)
Skin and SC tissue disorders	11 (91.7)	0 (0.0)	9 (90.0)	3 (30.0)	41 (95.3)	21 (48.8)	61 (93.8)	24 (36.9)
Dry skin	0 (0.0)	0 (0.0)	1 (10.0)	0 (0.0)	10 (23.3)	0 (0.0)	11 (16.9)	0 (0.0)
Pruritus	1 (8.3)	0 (0.0)	4 (40.0)	0 (0.0)	8 (18.6)	0 (0.0)	13 (20.0)	0 (0.0)
Rash maculo-papular	9 (75.0)	0 (0.0)	9 (90.0)	3 (30.0)	40 (93.0)	20 (46.5)	58 (89.2)	23 (35.4)
Adverse drug reactions								
Any events	11 (91.7)	3 (25.0)	10 (100.0)	6 (60.0)	43 (100.0)	30 (69.8)	64 (98.5)	39 (60.0)
GI disorders	4 (33.3)	0 (0.0)	8 (80.0)	0 (0.0)	29 (67.4)	1 (2.3)	41 (63.1)	1 (1.5)
Diarrhea	1 (8.3)	0 (0.0)	2 (20.0)	0 (0.0)	5 (11.6)	0 (0.0)	8 (12.3)	0 (0.0)
Nausea	1 (8.3)	0 (0.0)	3 (30.0)	0 (0.0)	12 (27.9)	0 (0.0)	16 (24.6)	0 (0.0)
Stomatitis	3 (25.0)	0 (0.0)	8 (80.0)	0 (0.0)	26 (60.5)	1 (2.3)	37 (56.9)	1 (1.5)
Vomiting	2 (16.7)	0 (0.0)	1 (10.0)	0 (0.0)	4 (9.3)	0 (0.0)	7 (10.8)	0 (0.0)
General disorders and administration site conditions	2 (16.7)	0 (0.0)	5 (50.0)	0 (0.0)	20 (46.5)	0 (0.0)	27 (41.5)	0 (0.0)
Pyrexia	1 (8.3)	0 (0.0)	4 (40.0)	0 (0.0)	12 (27.9)	0 (0.0)	17 (26.2)	0 (0.0)
Investigations	5 (41.7)	1 (8.3)	6 (60.0)	3 (30.0)	28 (65.1)	16 (37.2)	39 (60.0)	20 (30.8)
ALT increased	1 (8.3)	0 (0.0)	0 (0.0)	0 (0.0)	6 (14.0)	2 (4.7)	7 (10.8)	2 (3.1)
AST increased	1 (8.3)	0 (0.0)	1 (10.0)	0 (0.0)	6 (14.0)	0 (0.0)	8 (12.3)	0 (0.0)
GGT increased	1 (8.3)	0 (0.0)	2 (20.0)	1 (10.0)	5 (11.6)	4 (9.3)	8 (12.3)	5 (7.7)
Neutrophil count decreased	3 (25.0)	0 (0.0)	3 (30.0)	0 (0.0)	15 (34.9)	9 (20.9)	21 (32.3)	9 (13.8)
WBC count decreased	3 (25.0)	1 (8.3)	3 (30.0)	2 (20.0)	19 (44.2)	5 (11.6)	25 (38.5)	8 (12.3)
Blood AP increased	1 (8.3)	0 (0.0)	1 (10.0)	0 (0.0)	5 (11.6)	0 (0.0)	7 (10.8)	0 (0.0)
Metabolism and nutrition disorders	5 (41.7)	2 (16.7)	5 (50.0)	2 (20.0)	26 (60.5)	7 (16.3)	36 (55.4)	11 (16.9)
Hyperglycemia	4 (33.3)	2 (16.7)	3 (30.0)	2 (20.0)	23 (53.5)	7 (16.3)	30 (46.2)	11 (16.9)
Decreased appetite	2 (16.7)	0 (0.0)	5 (50.0)	0 (0.0)	3 (7.0)	0 (0.0)	10 (15.4)	0 (0.0)
Skin and SC tissue disorders	11 (91.7)	0 (0.0)	9 (90.0)	3 (30.0)	41 (95.3)	21 (48.8)	61 (93.8)	24 (36.9)
Dry skin	0 (0.0)	0 (0.0)	1 (10.0)	0 (0.0)	7 (16.3)	0 (0.0)	8 (12.3)	0 (0.0)
Pruritus	1 (8.3)	0 (0.0)	4 (40.0)	0 (0.0)	8 (18.6)	0 (0.0)	13 (20.0)	0 (0.0)
Rash maculo-papular	9 (75.0)	0 (0.0)	9 (90.0)	3 (30.0)	40 (93.0)	20 (46.5)	58 (89.2)	23 (35.4)

Data are presented as *n* (%)

*ALT* alanine aminotransferase, *AP* alkaline phosphatase, *AST* aspartate aminotransferase, *DEP* dose escalation phase, *GGT* gamma-glutamyl transferase *GI* gastrointestinal, *RMP* regimen modification phase, *SAP* safety assessment phase, *SC* subcutaneous, *WBC* white blood cell

### Pharmacokinetics

Following the Cycle 1 Day 1 dose, the terminal phase elimination half-life (t_1/2_) of TAS-117 ranged from 22 to 32 h, and the time to maximum plasma concentration (t_max_) ranged from 2 to 4 h, regardless of dose (Supplemental Table 1). The accumulation ratio of C_max_ and AUC from 0 to 24 h (AUC_0–24_) was approximately 3- to 4-fold after repeated QD dosing and approximately 2- to 3-fold after repeated intermittent dosing (Supplemental Table 1). The t_1/2_ data indicate that a steady state was reached by Day 8. The urinary excretion rate ranged from 8 to 11%. The mean plasma concentration–time profiles of TAS-117 are shown in Fig. 1a and 1b. The dose proportionality analysis for the 8, 16, 24, and 32 mg doses is shown in Fig. 1c. There was a dose proportional increase in C_max_, AUC up to the last observable concentration (AUC_last_), and AUC_0–24_ in the 8 to 32 mg dose range. The 95% confidence intervals (CIs) of intercepts of linear regression lines for C_max_, AUC_last_, and AUC_0-24_ contained 0, and the 95% CI of slopes of power regression lines contained 1, thus fulfilling the criteria for dose proportionality (Supplemental Table 2).

### Pharmacodynamics

**Fig. 2:**
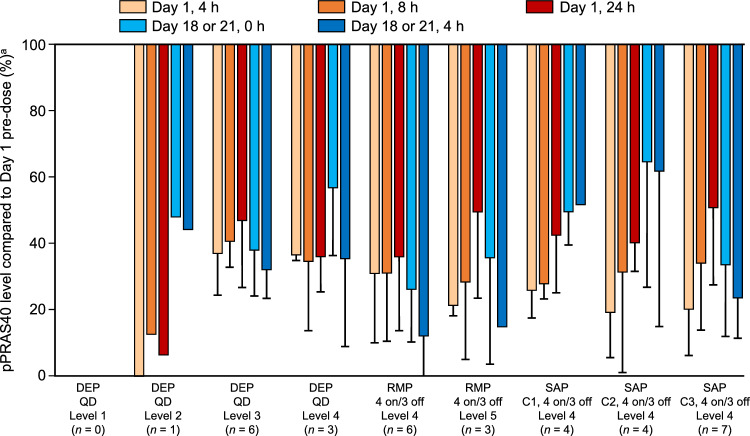
Mean changes in the ratio of pPRAS40/total PRAS40 after TAS-117 administration on Cycle 1 Day 1 compared with pre-dose. The phosphorylation status of PRAS40 in the blood was measured with an electrochemiluminescence detection assay and a Meso Scale Discovery instrument (Meso Scale Diagnostics, Rockville, MD, US). ^a^Ratio of pPRAS40/total PRAS40 (percentage compared with Day 1 pre-dose). *C* cohort, *DEP* dose escalation phase, *p* phosphorylated, *QD* once daily, *RMP* regimen modification phase, *SAP* safety assessment phase

The percentage change in pPRAS40/total PRAS40 values from before to after TAS-117 administration is shown in Fig. 2. After TAS-117 administration, a reduction in the level of pPRAS40 (> 50% at any time point) was observed in the DEP, RMP, and SAP for the range of TAS-117 dose levels 2 to 5 (8 to 32 mg/body/day).

### Efficacy

No patients in the DEP or RMP had a complete response (CR) or partial response (PR); in the SAP, no patients had CR, and 1 of 16 patients (6.3%) in cohort 1 and 3 of 20 patients (15.0%) in cohort 3 had confirmed PR (Table 3). However, the DCR was low for patients in the DEP or RMP (0 to 33.3%) except for dose level 2 in the DEP (100%). The DCR was 68.8%, 85.7%, and 40.0% for patients in cohort 1, cohort 2, and cohort 3, respectively, in the SAP.

**Table 3:** Best overall response

	DEP (QD)				RMP (intermittent dosing)		SAP (intermittent dosing)			Total
	Level 1 (*n* = 2)	Level 2 (*n* = 1)	Level 3 (*n* = 6)	Level 4 (*n* = 3)	Level 4 (*n* = 6)	Level 5 (*n* = 4)	Cohort 1 Level 4 (*n* = 16)	Cohort 2 Level 4 (*n* = 7)	Cohort 3 Level 4 (*n* = 20)	*N* = 65
CR	0	0	0	0	0	0	0	0	0	0
PR	0	0	0	0	0	0	1 (6.3)	0	3 (15.0)	4 (6.2)
SD	0	1 (100.0)	0	1 (33.3)	2 (33.3)	0	10 (62.5)	6 (85.7)	5 (25.0)	25 (38.5)
Non-CR/non-PD	0	0	0	0	0	0	0	0	0	0
PD	2 (100.0)	0	6 (100.0)	2 (66.7)	4 (66.7)	4 (100.0)	5 (31.3)	1 (14.3)	12 (60.0)	36 (55.4)
NE	0	0	0	0	0	0	0	0	0	0
RR (CR + PR)	0	0	0	0	0	0	1 (6.3)	0	3 (15.0)	4 (6.2)
95% CI, % (lower, upper)	0, 84.2	0, 97.5	0, 45.9	0, 70.8	0, 45.9	0, 60.2	0.2, 30.2	0, 41.0	3.2, 37.9	1.7, 15.0
DCR (CR + PR + SD + non-CR/non-PD)	0	1 (100.0)	0	1 (33.3)	2 (33.3)	0	11 (68.8)	6 (85.7)	8 (40.0)	29 (44.6)
95% CI, % (lower, upper)	0, 84.2	2.5, 100.0	0, 45.9	0.8, 90.6	4.3, 77.7	0, 60.2	41.3, 89.0	42.1, 99.6	19.1, 63.9	32.3, 57.5

Data are presented as *n* (%) unless otherwise indicated

*CI* confidence interval, *CR* complete response, *DCR* disease control rate, *DEP* dose escalation phase, *NE* not evaluable, *PD* progressive disease, *PR* partial response, *QD* once daily, *RMP* regimen modification phase, *RR* response rate, *SAP* safety assessment phase, *SD* stable disease

### Pharmacogenomics

**Fig. 3:**
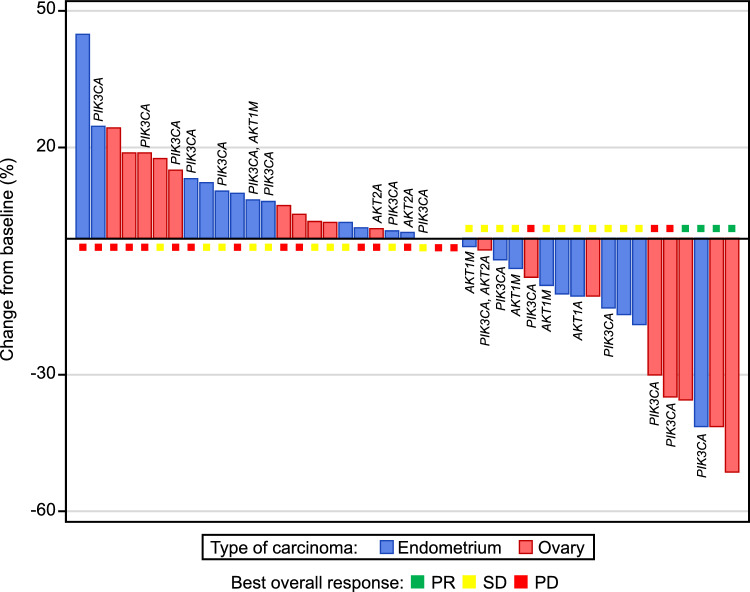
Maximum tumor change from baseline and pharmacogenomics (SAP). The status of *PIK3CA* mutation, *AKT1* mutation, and *AKT1* and *AKT2* amplification is displayed. Formalin-fixed paraffin-embedded tumor tissue specimens were tested at a central laboratory for the following: *PIK3CA*:*PIK3CA* mutation, *AKT1M*:*AKT1* mutation, *AKT1A*:*AKT1* amplification, and *AKT2A*:*AKT2* amplification. *PIK3CA* data were obtained using PCR, and *AKT1M*, *AKT1A*, and *AKT2A* data were obtained using droplet digital PCR. Endometrial cancer patients with unlabeled *PIK3CA* or *AKT* gene abnormality were tested by next-generation sequencing for tumor tissues, droplet digital PCR for blood samples, or local tests. *PCR* polymerase chain reaction, *PD* progressive disease, *PR* partial response, *SAP* safety assessment phase, *SD* stable disease

Pharmacogenomics results of *AKT* and *PIK3CA* gene abnormalities tested by PCR or droplet digital PCR in tumor tissue samples for patients in the SAP with endometrial cancer (cohorts 1 and 2; *n* = 23) and those with ovarian clear cell carcinoma (cohort 3; *n* = 20) are shown in Fig. 3. Given the lack of adequate clinical response in patients in the DEP, RMP, and cohorts 1 and 2 in the SAP, those groups were not considered suitable for examining predictive biomarkers. Among patients with ovarian clear cell carcinoma (cohort 3, SAP), 3 of 20 patients had PR; thus, candidate predictive biomarkers were analyzed in this population. However, no factors predictive of clinical response were identified.

## Discussion

The RD dosing for TAS-117 was determined as 16 mg per day for QD dosing and 24 mg per day for intermittent dosing in patients with advanced or metastatic solid tumors. TAS-117 demonstrated acceptable safety and pharmacokinetic profiles as well as inhibitory activity against AKT; anti-tumor activity was suggested in some patients.

Skin-related and gastrointestinal AEs, myelosuppression, and hyperglycemia were frequently observed with TAS-117 treatment. These AEs are known class side effects associated with PI3K/AKT/mTOR inhibitors [23]. Most patients (93.8%) developed AEs related to skin disorders; maculopapular rash was the most common (89.2%), followed by pruritis, dry skin, and rash. Maculopapular rash was grade ≥ 3 for 35.4% of patients with this AE. Skin-related AEs resolved on their own or with TAS-117 discontinuation or dose reduction, or with the administration of topical steroids, antihistamines, or oral steroids. The safety profile of TAS-117 was acceptable at doses up to 16 mg per day with QD dosing and up to 24 mg per day with intermittent dosing.

The pharmacokinetics of TAS-117 were dose proportional in the dosing range evaluated (8 to 32 mg). In the SAP, four patients (cohort 1 *PIK3CA* mutation-positive endometrial cancer, *n* = 1; cohort 3 ovarian clear cell carcinoma, *n* = 3) had confirmed PR, suggesting that TAS-117 has antitumor activity in patients with selected advanced solid cancers. Given the limited availability of effective treatment in ovarian clear cell carcinoma overall, this finding in three patients with ovarian clear cell carcinoma is worth further investigation. Ovarian clear cell carcinoma is relatively rare in the US and Europe (< 10% of ovarian cancers) [24, 25], but in Asian countries, the incidence rates are higher (13 to 25% of ovarian cancers) and have been increasing over time [26–28]. With later-stage disease, the response rate to conventional platinum-based therapy is lower compared with other major histological subtypes [29], and few clinical trials have specifically targeted ovarian clear cell carcinoma [30]. Thus, there is a great unmet need for effective treatment strategies for these patients.

Although the efficacy findings of this study are encouraging, this first-in-human study was limited by the small number of participants and the absence of a control group. Further studies investigating TAS-117 as monotherapy, and in combination with other approved oncology drugs, in larger patient populations are needed.

In conclusion, this study demonstrated that TAS-117 was well tolerated at doses up to 16 mg/day with QD dosing and at intermittent doses of 24 mg/day (4 days on/3 days off). All AEs were manageable by dose interruption, dose reduction, or targeted therapy. TAS-117 pharmacokinetics were dose proportional in the doses evaluated and its pharmacodynamics suggest that TAS-117 exhibits inhibitory activity against AKT in humans. Altogether with the antitumor activity findings, these findings suggest that TAS-117 exhibits antitumor activity by inhibiting AKT.

### Supplementary Information

Below is the link to the electronic supplementary material.

Supplementary file1 (PPTX 48 KB)

Supplementary file2 (DOCX 46 KB)

Supplementary file3 (DOCX 36 KB)

Supplementary file4 (DOCX 36 KB)

## Data Availability

Data will not be shared according to the Sponsor’s policy on data sharing, which can be found at: https://www.taiho.co.jp/en/science/policy/clinical_trial_information_disclosure_policy/.
