# Supplementary material for: A first-in-human phase I study of TAS-117, an allosteric AKT inhibitor, in patients with advanced solid tumors
Source: Cancer Chemother Pharmacol. 2024 Feb 27;93(6):605–16. doi: 10.1007/s00280-023-04631-7 (PMC11129975; doi:10.1007/s00280-023-04631-7)
Supplement: Supplementary file 1 — Supplementary file1 (PPTX 48 KB) [file 280_2023_4631_MOESM1_ESM.pptx]

## Slide 1
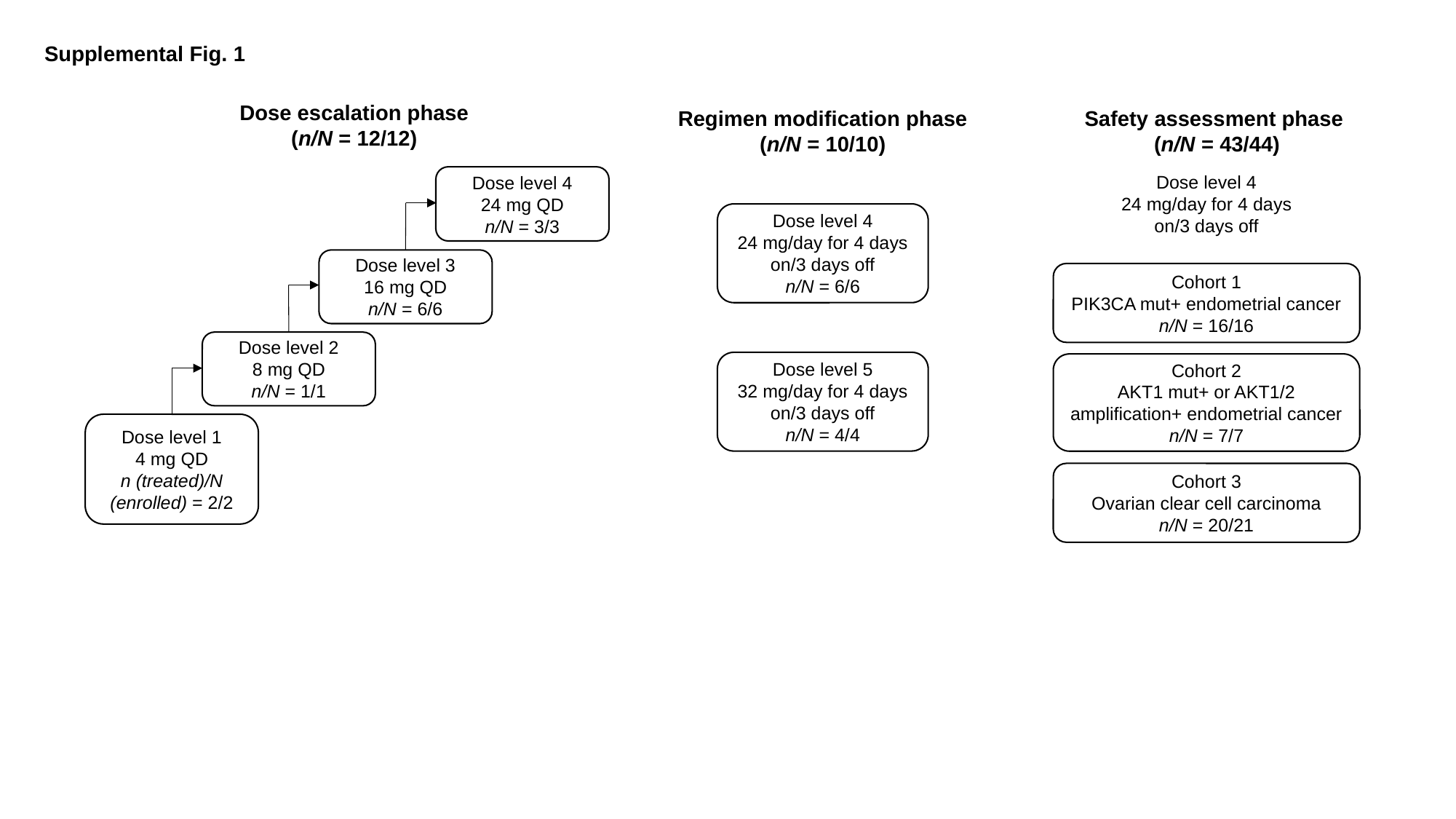

Supplemental Fig. 1
Dose escalation phase
(n/N = 12/12)
Regimen modification phase
(n/N = 10/10)
Safety assessment phase
(n/N = 43/44)
Dose level 4
24 mg/day for 4 days on/3 days off
Dose level 4
24 mg QD
n/N = 3/3
Dose level 4
24 mg/day for 4 days on/3 days off
n/N = 6/6
Dose level 3
16 mg QD
n/N = 6/6
Cohort 1
PIK3CA mut+ endometrial cancer
n/N = 16/16
Dose level 2
8 mg QD
n/N = 1/1
Dose level 5
32 mg/day for 4 days on/3 days off
n/N = 4/4
Cohort 2
AKT1 mut+ or AKT1/2 amplification+ endometrial cancer
n/N = 7/7
Dose level 1
4 mg QD
n (treated)/N (enrolled) = 2/2
Cohort 3
Ovarian clear cell carcinoma
n/N = 20/21
