# Supplementary material for: A first-in-human phase I study of TAS-117, an allosteric AKT inhibitor, in patients with advanced solid tumors
Source: Cancer Chemother Pharmacol. 2024 Feb 27;93(6):605–16. doi: 10.1007/s00280-023-04631-7 (PMC11129975; doi:10.1007/s00280-023-04631-7)
Supplement: Supplementary file 2 — Supplementary file2 (DOCX 46 KB) [file 280_2023_4631_MOESM2_ESM.docx]

**Supplemental Table 1** Pharmacokinetics of TAS-117

|  | **C_max_**  **(ng/mL)** | **t_max_**  **(h)** | **AUC_last_**  **(ng·hr/mL)** | **AUC_0–24_**  **(ng·hr/mL)** | **t_1/2_**  **(h)** | **Fe**  **(%)** | **CLr**  **(L/h)** |
| --- | --- | --- | --- | --- | --- | --- | --- |
| DEP |  |  |  |  |  |  |  |
| Cycle 1 Day 1 |  |  |  |  |  |  |  |
| 8 mg QD, *n* = 1 | 8.82 | 1.93 | 165 | 164 | 21.96 | 8.45 | 4.11 |
| 16 mg QD, *n* = 6 |  |  |  |  |  |  |  |
| Mean (SD) | 25.1 (7.66) | 2.98 (1.88, 3.89)^c^ | 316 (73) | 315 (73) | 26.98 (6.06) | 10.43 (4.94) | 5.20 (2.19) |
| CV% | 30.6 | – | 23.3 | 23.2 | 22.5 | 47.4 | 42.1 |
| gMean | 24.1 | – | 308 | 308 | 26.40 | 9.20 | 4.78 |
| gCV% | 32.3 | – | 24.7 | 24.6 | 23.3 | 65.3 | 49.8 |
| 24 mg QD, *n* = 3 |  |  |  |  |  |  |  |
| Mean (SD) | 31.0 (15.7) | 3.98 (3.92, 6.00)^c^ | 438 (193) | 438 (194) | 23.06 (NC)^d^ | 10.48 (2.73) | 6.39 (2.72) |
| CV% | 50.6 | – | 43.9 | 44.2 | NC | 26.0 | 42.6 |
| gMean | 28.6 | – | 408 | 407 | 22.61^d^ | 10.25 | 6.04 |
| gCV% | 51.0 | – | 50.2 | 50.8 | NC | 25.6 | 41.4 |
| Cycle 1 Day 21 |  |  |  |  |  |  |  |
| 8 mg QD, *n* = 1 | 33.1 | 7.85 | 604 | NC | NC | – | – |
| 16 mg QD, *n* = 3 |  |  |  |  |  |  |  |
| Mean (SD) | 61.4 (22.2) | 1.93 (1.87, 2.02)^c^ | 864 (220) | 865 (216) | 41.65 (NC)^d^ | – | – |
| CV% | 36.2 | – | 25.4 | 25.0 | NC | – | – |
| gMean | 58.3 | – | 844 | 846 | 41.23^d^ | – | – |
| gCV% | 42.6 | – | 27.9 | 27.3 | NC | – | – |
| 24 mg QD, *n* = 0 |  |  |  |  |  |  |  |
| Observed accumulation ratio |  |  |  |  |  |  |  |
| 8 mg QD, *n* = 1 | 3.75 | – | – | NC | – | – | – |
| 16 mg QD, *n* = 3 |  |  |  |  |  |  |  |
| Mean (SD) | 2.71 (0.48) | – | – | 2.84 (0.04) | – | – | – |
| CV% | 17.7 | – | – | 1.3 | – | – | – |
| gMean | 2.69 | – | – | 2.83 | – | – | – |
| gCV% | 17.5 | – | – | 1.3 | – | – | – |
| 24 mg QD, *n* = 0 |  |  |  |  |  |  |  |
| RMP |  |  |  |  |  |  |  |
| Cycle 1 Day 1 |  |  |  |  |  |  |  |
| 24 mg intermittent dosing^a^, *n* = 6 |  |  |  |  |  |  |  |
| Mean (SD) | 42.1 (11.1) | 3.28 (0.95, 4.15)^c^ | 542 (107) | 543 (108) | 31.92 (10.77)^e^ | 10.02 (2.85) | 4.55 (1.43) |
| CV% | 26.3 | – | 19.7 | 19.9 | 33.8^e^ | 28.4 | 31.4 |
| gMean | 40.9 | – | 533 | 534 | 30.44^e^ | 9.65 | 4.34 |
| gCV% | 26.6 | – | 19.8 | 20.0 | 35.8^e^ | 31.1 | 36.3 |
| 32 mg intermittent dosing^b^, *n* = 4 |  |  |  |  |  |  |  |
| Mean (SD) | 60 (18.4) | 2.43 (1.95, 4.03)^c^ | 774 (162) | 770 (155) | 24.02 (3.80) | 9.32 (2.71) | 4.12 (1.82) |
| CV% | 30.7 | – | 20.9 | 20.2 | 15.8 | 29.1 | 44.2 |
| gMean | 58.0 | – | 761 | 758 | 23.81 | 9.01 | 3.80 |
| gCV% | 30.3 | – | 21.4 | 20.6 | 15.2 | 30.9 | 49.9 |
| Cycle 1 Day 18 |  |  |  |  |  |  |  |
| 24 mg intermittent dosing^a^, *n* = 5 |  |  |  |  |  |  |  |
| Mean (SD) | 89.9 (37.4) | 2.00 (0.98, 5.92)^c^ | 3542 (1023) | 1498 (500) | 49.31 (7.73) | – | – |
| CV% | 41.6 | – | 28.9 | 33.4 | 15.7 | – | – |
| gMean | 83.8 | – | 3420 | 1433 | 48.83 | – | – |
| gCV% | 43.9 | – | 30.7 | 34.1 | 15.5 | – | – |
| 32 mg intermittent dosing^b^, *n* = 1 | 160 | 1.00 | 5558 | 2105 | 49.63 | – | – |
| Observed accumulation ratio |  |  |  |  |  |  |  |
| 24 mg intermittent dosing^a^, *n* = 5 |  |  |  |  |  |  |  |
| Mean (SD) | 2.06 (0.73) | – | – | 2.70 (0.37) | – | – | – |
| CV% | 35.5 | – | – | 13.8 | – | – | – |
| gMean | 1.95 | – | – | 2.68 | – | – | – |
| gCV% | 39.7 | – | – | 14.2 | – | – | – |
| 32 mg intermittent dosing^b^, *n* = 1 | 2.50 | – | – | 2.69 | – | – | – |

Patients with dose modifications or dose interruptions were excluded from the analysis after multiple doses.

^a^24 mg/day for 4 days on/3 days off

^b^32 mg/day for 4 days on/3 days off

^c^Data are presented as median (min, max)

^d^*n* = 2

^e^*n* = 5

*AUC* area under the plasma concentration-time curve, *AUC_0–24_* AUC from time 0 to 24 h, *AUC_last_* AUC up to the last observable concentration, *CLr* renal clearance, *C_max_* maximum plasma concentration, *CV* coefficient of variation, *DEP* dose escalation phase, *Fe* urinary excretion rate as percentage of dose, *gCV* geometric coefficient of variation, *gMean* geometric mean, *NC* not calculated, *QD* once daily, *RMP* regimen modification phase, *SD* standard deviation *t_1/2_* terminal phase elimination half-life, *t_max_* time to maximum plasma concentration
