# Supplementary material for: A first-in-human phase I study of TAS-117, an allosteric AKT inhibitor, in patients with advanced solid tumors
Source: Cancer Chemother Pharmacol. 2024 Feb 27;93(6):605–16. doi: 10.1007/s00280-023-04631-7 (PMC11129975; doi:10.1007/s00280-023-04631-7)
Supplement: Supplementary file 3 — Supplementary file3 (DOCX 36 KB) [file 280_2023_4631_MOESM3_ESM.docx]

**Supplemental Table 2** Dose proportionality analysis (pharmacokinetic parameters on Cycle 1, Day 1)

|  | **C_max_** | **AUC_last_** | **AUC_0–24_** |
| --- | --- | --- | --- |
| Linear regression analysis |  |  |  |
| Observations (*n*) | 63 | 63 | 63 |
| Intercept estimate | –9.48 | –103.93 | –101.86 |
| Slope estimate | 2.13 | 28.52 | 28.41 |
| 95% CI for intercept (lower, upper) | (–30.30, 11.35) | (–348.49, 140.63) | (–348.29, 144.57) |
| *p*-value^a^ | 0.97 | 0.71 | 0.69 |
| Power regression analysis |  |  |  |
| Observations (*n*) | 63 | 63 | 63 |
| Intercept estimate | –0.48 | 2.36 | 2.36 |
| Slope estimate | 1.31 | 1.25 | 1.25 |
| 95% CI for slope (lower, upper) | (0.91, 1.71) | (0.92, 1.58) | (0.92, 1.58) |
| *p*-value^a^ | 0.95 | 0.52 | 0.52 |

^a^*P*-values were calculated for lack-of-fit testing.

*AUC* area under the plasma concentration time curve, *AUC_0–24_* AUC from time 0 to 24 h, *AUC_last_* AUC up to the last observable concentration, *C_max_* maximum plasma concentration, *CI* confidence interval
