# Supplementary material for: A first-in-human phase I study of TAS-117, an allosteric AKT inhibitor, in patients with advanced solid tumors
Source: Cancer Chemother Pharmacol. 2024 Feb 27;93(6):605–16. doi: 10.1007/s00280-023-04631-7 (PMC11129975; doi:10.1007/s00280-023-04631-7)
Supplement: Supplementary file 4 — Supplementary file4 (DOCX 36 KB) [file 280_2023_4631_MOESM4_ESM.docx]

***Supplemental Text***

***Supplemental Text 1.*** Full exclusion criteria.

A patient was excluded from the study if any of the following conditions were observed:

1. History or current evidence of type 1 or type 2 diabetes mellitus that requires insulin and/or oral antidiabetic therapy.
2. Current evidence of retinopathy that requires ophthalmological therapy.
3. History or current evidence of cardiac arrhythmia and/or conduction abnormality.
4. Treatment with any of the following within the specified time frame prior to study drug administration:
   1. Major surgery within the prior 4 weeks (the surgical incision should be fully healed prior to study drug administration).
   2. Radiation therapy for extended field within 4 weeks prior to study drug administration or limited field radiation therapy within 2 weeks prior to study drug administration.
   3. Any anticancer treatment within 3 weeks prior to study drug administration (mitomycin within the prior 5 weeks).
   4. Any investigational agent received either concurrently or within the last 3 weeks.
5. A serious illness of medical condition(s) including, but not limited to, the following:
   1. Known brain metastasis not stable off steroids for ≥2 months.
   2. Known leptomeningeal metastasis.
   3. Known acute systemic infection.
   4. Myocardial infarction, severe/unstable angina, symptomatic congestive heart failure (New York Heart Association class III or IV within the previous 6 months; if >6 months cardiac function must be within normal limits and the patient must be free of cardiac-related symptoms).
   5. Chronic nausea or diarrhea considered to be clinically significant in the opinion of the Investigator or Sub-investigator.
   6. Known severe chronic kidney disease.
   7. Known human immunodeficiency virus or acquired immunodeficiency syndrome-related illness, or a history of serum positivity to hepatitis B or C.
   8. History or current evidence of interstitial lung disease that requires steroid medication.
   9. Other severe acute or chronic medical or psychiatric condition or laboratory abnormality that may increase the risk associated with study participation or study drug administration, or may interfere with the interpretation of study results, and in the judgment of the Investigator or Sub-investigator would make the patient inappropriate for entry into this study.
6. Unresolved toxicity of Grade >1 attributed to any prior therapies (excluding alopecia, skin pigmentation, and anemia).
7. Patients with the risk of hypokalemia (e.g., receiving high dose diuretic therapy).
8. Receiving oral steroid medication.
9. Known hypersensitivity to any drugs similar to TAS-117 in structure or class.
10. Prior therapy with TAS-117.
11. Pregnant or lactating female (including the cessation of lactation) or females of childbearing potential who have a positive pregnancy test (urine or serum) within 7 days prior to the day on which the study drug is scheduled to be administered. Males and females who do not agree to adequate birth control if conception is possible during the clinical study and for 180 days after the last dose. Female patients are considered not to be of child-bearing potential if they have a history of tubal ligation or hysterectomy or are post-menopausal with a minimum of 1 year without menses (excluding patients with lack of menses due to medical reasons, e.g., receipt of medication such as contraception and anticancer treatment).
12. In the safety assessment phase, patients who have other primary malignancies other than carcinomas in situ, e.g., breast, cervix, and prostate.
